# Cytologic evaluation of image-guided fine needle aspiration biopsies via robotic microscopy: A validation study

**DOI:** 10.4103/2153-3539.63826

**Published:** 2010-05-26

**Authors:** Guoping Cai, Lisa A. Teot, Walid E Khalbuss, Jing Yu, Sara E. Monaco, Drazen M. Jukic, Anil V. Parwani

**Affiliations:** 1 Department of Pathology, University of Pittsburgh Medical Center, Pittsburgh, PA Yale University School of Medicine, New Haven, CT,USA; 2 Department of Pathology, Yale University School of Medicine, New Haven, CT,USA

**Keywords:** Fine needle aspiration biopsy, robotic microscopy, telecytology

## Abstract

**Background:**

This study carried out was to assess the feasibility of using robotic microscopy (RM) for cytologic evaluation of direct smears from fine needle aspiration biopsy (FNAB).

**Methods:**

Three board-certified cytopathologists reviewed representative direct smears from 40 image-guided FNABs using RM and subsequently re-reviewed the same smears using conventional microscopy. Adequacy of the smears and cytologic diagnosis, as determined using the two approaches, were compared for each individual cytopathologist (intraobserver) and between the three cytopathologists (interobserver). The intraobserver and interobserver discrepancies were analyzed and discussed in a follow-up consensus conference.

**Results:**

For assessment of adequacy, there were high concordance rates (intraobserver: 92.5–97.5%; interobserver: 90–92.5%), with a few discrepancies involving distinctions between suboptimal and satisfactory smears. Analysis of diagnostic interpretations showed correct classification of 92.5–95% (intraobserver) or 90–92.5% (interobserver) of benign and malignant cases combined, with the discrepancies being between benign and atypical cells in the benign group, and between suspicious and malignant in the malignant group. Within the malignant group, 94% of cases were accurately subclassified via RM. The quality of images viewed by using RM was rated adequate (fair or good) for 95% of the slides.

**Conclusions:**

The results demonstrate that cytologic evaluation of direct smears from FNABs using RM is feasible. Problems encountered included the longer times needed to evaluate cases with thick, bloody smears and/or low numbers of diagnostic cells, and difficulties in recognizing neuroendocrine differentiation and mimics of hepatocellular carcinoma.

## INTRODUCTION

With the advancement of the field of digital pathology from static images to dynamic imaging and virtual microscopy,[1,2] robotic microscopy (RM) and whole slide imaging (WSI) are gaining popularity as tools for diagnosis, education, and research. The former provides real-time images via a remotely robot-controlled microscope, whereas the latter acquires permanent images of entire slides using automated high-resolution scanners. Previous studies have demonstrated the validity of these techniques in clinical practices.[3–12] RM- or WSI-based telepathology is being integrated into various aspects of surgical pathology, including intraoperative diagnosis, consultation services, quality control, education, teaching, and research.[4,5,7,9–16] In addition, the American Board of Pathology is experimenting with virtual microscopy for a small portion of the anatomic pathology examination. Recently, the WSI-based approach has also been used for cytologic evaluations.[17–19] 

Fine needle aspiration biopsy (FNAB) has gained wide acceptance as a diagnostic tool in the clinical management of palpable and nonpalpable masses. A key component of FNAB is the on-site, immediate assessment which ensures adequate sampling, guides appropriate triage of the specimen for ancillary studies, and in some cases, allows a preliminary diagnosis that may expedite clinical management and treatment. Because on-site evaluation requires the physical presence of a cytopathologist, it may be difficult to incorporate into a busy cytology service, given that each image-guided procedure takes approximately 45–55 minutes.[20] RM has the potential for enabling “on-site” evaluations while decreasing the time a cytopathologist spends on each procedure. In this pilot study, we examined the feasibility of using RM for cytologic evaluation of direct smears from image-guided FNABs.

## METHODS

### Case Selection

A total of 40 cases of image-guided FNABs were retrieved from the cytopathology archives of the University of Pittsburgh Medical Center Shadyside, Pittsburg, PA, USA. The cases included FNABs of the lung (20 cases), liver (16 cases), mesentery (2 cases), pleura (1 case), and kidney (1 case). The final categorical diagnosis was malignant in 35 cases (87.5%) and benign in 5 cases (12.5%). The FNABs were performed using 25-gauge spinal needles under the guidance of CT (22 cases) or ultrasound (18 cases). Two representative smears (one air-dried, Diff-Quik-stained and one alcohol-fixed, Papanicolaou-stained) were selected from each case for review. The slides were deidentified and a new numerical code was assigned to each slide. Data provided to the reviewers included the patient's age and gender, anatomic site, and a brief clinical history. This study was approved by the University of Pittsburgh Institutional Review Board and was carried out at the University of Pittsburgh Medical Center Shadyside.

### Digital Pathology System: Robotic Microscopy and Associated Workflow

The system included a network-connected, fully robotic microscope and an attached 50-slide storage box (Microscope: Olympus BX51 (Olympus America Inc., Center Valley, PA, USA); System: Trestle SL-50 (Trestle Corporation, Newport Beach, CA, USA); Workstation: Dell Precision (Dell Inc., Round Rock, TX, USA)). The deidentified, coded slides were preloaded into a network accessible remote graphic user interface, MedMicroscopy (Trestle Corporation, Newport Beach, CA, USA), which created a “thumbnail” overview of all slides in the storage box. The robotic microscope system supports remote operations by allowing full remote access to the functions of a microscope, including high-resolution digital imagery from the connected network computers via a web-based communication. The system allows fully remote slide access, including barcode recognition with seamless integration of existing laboratory information systems. The system also has features such as rapid whole slide overview scanning for navigation, label imagery for verification and annotation. Automatic slide changing is also supported, which allows true remote reading of cases with multiple smears from a single pass or multiple passes. Up to 128 simultaneous users can be connected to a single robotic microscope, allowing a virtual multiheaded consultation.

Selection of slides for viewing was by the newly assigned numerical code. Once the slide of interest was loaded, it was viewed via the internet at the viewing sites with a high-resolution live view (1024X768 at 24 bit) through the MedMicroscopy application. No color correction except for white balance at the viewing sites was performed. Through the internet accessible control system, the cytopathologists were able to remotely control slide loading and microscope operations such as navigating the slide (*X, Y* axes), changing objective lenses, and adjusting lighting, contrast, and focus (Z axis). Workflow enhancing functions such as AutoFocus were also provided. In addition, the system also permitted digital photography of the case by the cytopathologist. The overall quality of the RM images was assessed and rated as poor, fair, or good for each smear by each cytopathologist independently.

### Cytologic Evaluation

The slides were evaluated independently by three board-certified cytopathologists (GC, WK, LT) first by RM and later by conventional light microscopy. Evaluation of each case included assessment of adequacy and a cytologic diagnosis. Adequacy was rated as satisfactory, suboptimal, or unsatisfactory. The cytologic diagnoses were rendered solely based on cytomorphologic analysis in an effort to simulate an on-site assessment more closely. The cases were first classified as negative (benign), atypical, suspicious for malignancy, or malignant (categorical diagnosis). If a malignant diagnosis was rendered, further classification such as carcinoma, sarcoma, melanoma, or neuroendocrine tumor or carcinoma was pursued. The study diagnoses were compared to the original final diagnoses, which were based on cytomorphologic analysis of glass slides and, if appropriate, ancillary studies.

### Data Analysis

To assess the feasibility of remote robotic microscopic evaluation of direct smears from FNABs, the concordance rates between the RM and conventional microscopic evaluation (intraobserver correlation rate) and those among the participating cytopathologists (interobserver correlation rate) were calculated. The concordance rates covered assessment of sample adequacy and categorical cytologic diagnosis. The cases that showed discordance were reviewed in a consensus conference and the reasons for the discordance were analyzed. The correlation between the RM and conventional microscopic evaluation for further classifying the malignant cells was also reviewed.

## RESULTS

The participating cytopathologists were generally satisfied with the overall quality of the images by RM. The quality was rated as adequate (fair or good) in 87.5, 97.5, and 100% of cases by cytopathologists A, B, and C, respectively [Table 1]. Five cases (12.5%) were rated as poor by one cytopathologist and one case (2.5%) rated as poor by another cytopathologist. Smears for images that were rated as poor were all thick and/or bloody. 

**Table 1:** Assessment of the quality of images of image-guided fine needle aspiration biopsies using robotic microscopy verses conventional microscopy

	Total cases	Robotic microscopy			Conventional microscopy		
		Good	Fair	Poor	Good	Fair	Poor
CP-A	40	22	13	5	35	5	0
CP-B	40	21	18	1	29	11	0
CP-C	40	34	6	0	40	0	0

CP: Cytopathologist

 Of the 40 cases, 37 (92.5%) or 38 (95%) cases were assessed as satisfactory for cytologic evaluation by RM [Table 2]. The remaining three or two cases were rated as less than optimal for evaluation due to low cellularity, obscuring blood, or suboptimal slide preparation. No case was considered unsatisfactory. There was a high concordance rate for adequacy assessment between the cytopathologists (interobserver correlation rate: 90–92.5%). For the individual cytopathologists, the concordance rates for adequacy assessment by RM and conventional slide review were 97.5–100% (intraobserver correlation rate) [Table 2]. The interobserver and intraobserver discrepancies were minor, involving satisfactory verses less than optimal ratings.

**Table 2:** Assessment of the quality of images of image-guided fine needle aspiration biopsies using robotic microscopy verses conventional microscopy

	Total cases	Robotic microscopy			Conventional microscopy		
		SAT	LTO	UNSAT	SAT	LTO	UNSAT
CP-A	40	37	3	0	37	3	0
CP-B	40	38	2	0	37	3	0
CP-C	40	38	2	0	37	3	0

CP: Cytopathologist; SAT: Satisfactory; LTO: Less than optimal; UNSAT: Unsatisfactory

For the categorical cytologic diagnosis, the malignant tumors were recognized in 94.3–100% of cases when reviewed by RM [Table 3]. Either one or two malignant cases (2.9 or 5.7%) were classified as suspicious for malignancy by two of the participating cytopathologists. Conventional microscopic evaluation yielded similar results for the categorical diagnosis of malignant cases by individual cytopathologists [Table 3]. The concordance rates for malignant cases between the two approaches were 97.5–100% within the individual cytopathologists (intraobserver variation). For the benign cases, 40–80% of the cases were rated as benign with the remainder rated as atypical (20–60%) by both the methods. Although there were discrepancies in benign and atypical diagnoses among the cytopathologists, particularly while using RM (interobserver variation), benign and atypical diagnoses were more consistent within the individual cytopathologists between the two approaches (intraobserver concordance rates, 80–100%).

**Table 3:** Categorical cytologic diagnoses of image-guided fine needle aspiration biopsies using robotic microscopy verses conventional microscopy

	Total cases	Robotic microscopy				Conventional slide review			
		Benign	Atypical	Susp	Malign	Benign	Atypical	Susp	Malign
CP-A	40	3	2	2	33	4	1	1	34
CP-B	40	4	1	0	35	4	1	1	34
CP-C	40	2	3	1	34	2	3	1	34

CP: Cytopathologist; susp, suspicious for malignancy; malign, positive for malignancy

The two cases that were called “suspicious for malignancy” by RM included one case each of squamous cell carcinoma and adenocarcinoma of the lung. In addition, one case was classified as suspicious for malignancy by all three cytologists using conventional microscopic evaluation. The main contributing factor for the less definitive diagnosis was a paucity of diagnostic cells on the smears. In the benign cases, the cases with “atypical” diagnoses included two lung biopsies and one liver biopsy.

**Figure 1:**
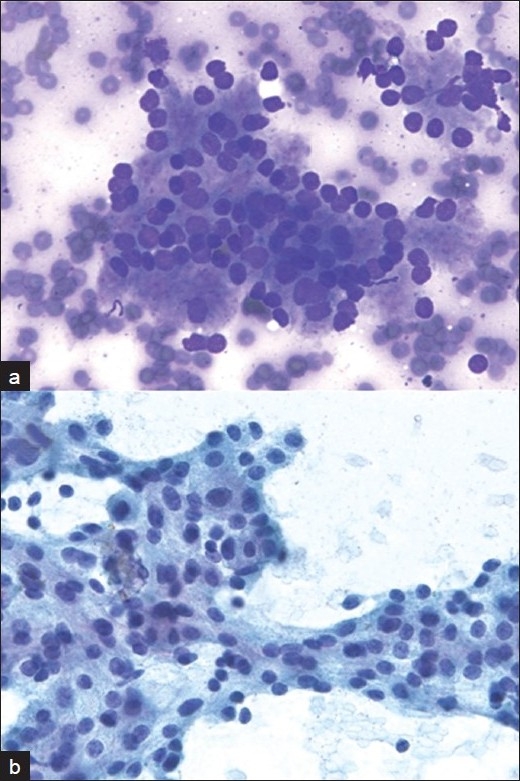
Robotic microscopic photographs of carcinoid tumor in the lung. The smears show clusters of relatively uniform tumor cells with vague acinar formation (a: Diff-Quik stain, ×400) and round to oval nuclei with speckled chromatin and inconspicuous nucleoli (b: Papanicolaou stain, ×400). This case was interpreted as non-neuroendocrine carcinoma by one cytopathologist

**Figure 2:**
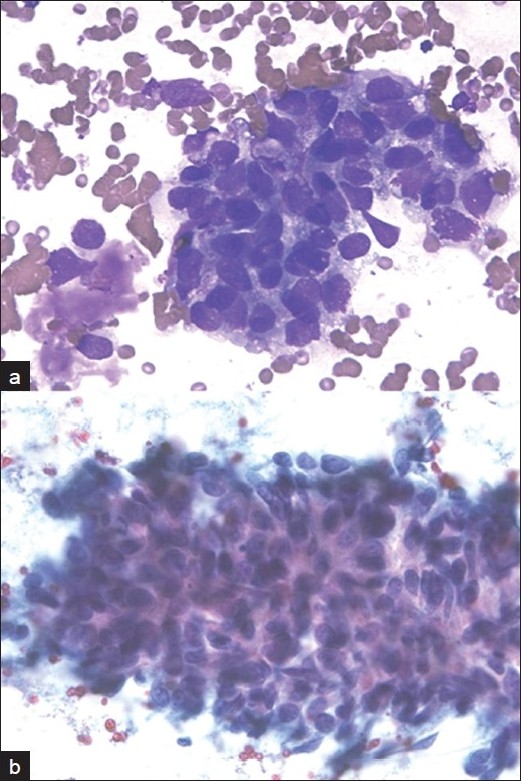
Robotic microscopic photographs of metastatic adenocarcinoma in the liver. The smears show clusters of malignant epithelial cells with high nuclear to cytoplasmic ratios, vacuolated cytoplasm (A: Diff-Quik stain, ×400) and hyperchromatic nuclei with small conspicuous nucleoli (B: Papanicolaou stain, ×400). This case was interpreted as hepatocellular carcinoma by one cytopathologist

**Figure 3:**
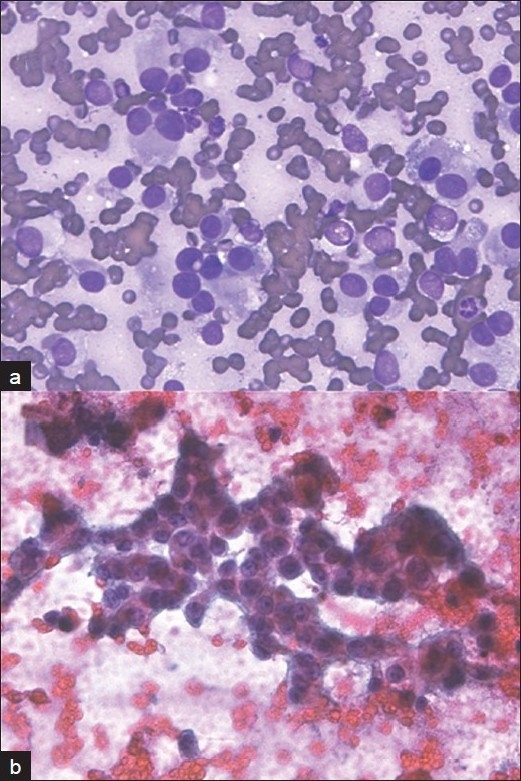
Robotic microscopic photographs of metastatic melanoma in the liver. The smears show single (A: Diff-Quik stain, ×400) and clusters of tumor cells (B: Papanicolaou stain, ×400) with abundant cytoplasm and round nuclei with prominent nucleoli. This case was interpreted as hepatocellular carcinoma by one cytopathologist

In most cases, the malignant tumors were accurately subclassified using RM as non-small cell carcinoma, adenocarcinoma, squamous cell carcinoma, carcinoma not otherwise specified, renal cell carcinoma, and sarcoma [Table 4]. However, there were discrepancies among the participating cytopathologists regarding the cytomorphologic diagnosis of neuroendocrine carcinoma and mimics of hepatocellular carcinoma. Three neuroendocrine tumors were not recognized [Figure 1], whereas one non-small cell carcinoma was misclassified as neuroendocrine carcinoma by two cytopathologists. Two adenocarcinomas and one melanoma were interpreted as hepatocellular carcinoma by one or two cytopathologists [Figures 2 and 3].

**Table 4:** Malignant cytologic diagnoses of image-guided fine needle aspiration biopsies using robotic microscopy verses conventional microscopy

	Robotic microscopy					Conventional microscopy				
	Total cases	MAL	NE	HCC	Mel	Total cases	MAL	NE	HCC	MEL
CP-A	33	23	6	2	2	34	23	6	2	2
CP-B	35	27	5	1	2	34	25	5	1	2
CP-C	34	27	3	3	1	34	26	4	2	1

CP: Cytopathologist; MAL: All malignant tumors except for neuroendocrine carcinoma, hepatocellular carcinoma, and melanoma; NE: Neuroendocrine carcinoma; HCC: Hepatocellular carcinoma; MEL: Melanoma

## DISCUSSION

Telepathology, in the forms of static images and virtual microscopy, has recently been utilized for the evaluation of cytologic preparations such as direct smears and monolayered slides.[17–19,21–27] However, evaluation of cytologic specimens by these methods appears to be less successful as compared to evaluation of surgical specimens.[18,19,23,26] Several factors may compromise the accurate assessment of cytologic specimens. In contrast to the two-dimensional histologic sections in surgical specimens, the cells and cellular aggregates in cytologic preparations are three-dimensional. Cytologic preparations may also be obscured by blood and/or the cells of interest may be entrapped within blood clots. The cellularity in cytologic preparations may be low due to dilution by blood or other background material, or simply due to sampling. These characteristics of cytologic preparations require constant refocusing of the microscope to fully evaluate the cytomorphologic features, a factor that cannot be accommodated with static images or virtual microscopy at present. Furthermore, static images and virtual microscopy are unable to deliver a real-time analysis and may hamper the rapid communication between the performers and the interpreters of FNABs.

The RM employed in the current study is a telepathology system that allows full remote control of the microscope including adjustment of focus. The quality of images viewed by RM was considered adequate by the participating cytopathologists. Direct smears from FNABs were successfully evaluated with RM for adequacy and cytologic diagnosis with high interobserver concordance rates. Moreover, interpretations rendered by RM and conventional light microscopy were very similar (high intraobserver concordance rates).

Only a few cases showed discordance in the categorical cytologic diagnoses. The differences in interpretation were minor, with the malignant tumors being interpreted as “suspicious for malignancy” and the benign cases being interpreted as “atypical cells”. Further analysis revealed that the discordance in malignant cases was mainly an intraobserver issue and the cases with discordance were those having low cellularity. In contrast, the discordance in the benign cases was mainly an interobserver issue and is attributed to subjective variations in interpretation.

In this study, accurate subclassification of most malignant tumors was possible based on the cytomorphologic features alone. The cases that posed diagnostic challenges included neuroendocrine carcinomas and mimics of hepatocellular carcinomas. As shown in Table 3, three of five neuroendocrine tumors were misclassified as non-small cell carcinoma by one cytopathologist, whereas one non-small cell carcinoma was misclassified as neuroendocrine carcinoma by another cytopathologist. This discrepancy appears to be due, in part, to one cytopathologist's diagnostic criteria because two of the three cases of misclassified neuroendocrine carcinoma were interpreted as non-small cell carcinoma by both RM and conventional microscopy. There might also be a systemic error that compromises recognition of nuclear/chromatin features of neuroendocrine tumors. Further study involving more cases of endocrine tumors to clarify the presence or absence of such a systemic error may be warranted. Misdiagnosis of mimics of hepatocellular carcinoma also appeared to be an interpretative issue. Three malignancies were misclassified as hepatocellular carcinoma by one or two participating cytopathologists using RM and two of those cases were also misclassified as hepatocellular carcinoma using conventional microscopy. 

Although the exact times were not recorded for evaluation of each case in the current study, there was a general impression that RM required a longer time in some cases as compared to the conventional microscopy. The cases in which this subjective difference was most noticeable were those with obscuring blood or low cellularity. This finding is similar to a previous study employing virtual microscopy.[19] Despite this limitation, the adoption of RM for remote evaluation of FNAB has the potential to save significant amounts of time by eliminating the time spent traveling to and from various sites and waiting between passes, and almost certainly offers a considerable improvement as compared to the previously reported 45–55 minutes per FNAB procedure needed for physical on-site evaluation.[20] 

Although this study demonstrates that use of RM for evaluation of direct smears from image-guided FNABs is feasible, additional studies are needed before incorporating this system into the daily practice of cytopathology. An additional obstacle to implementation of RM for remote on-site evaluation is the cost of the system. Analysis of the cost effectiveness of RM is needed, as is the analysis of impact on patient care, particularly in settings where on-site evaluations are not part of current practice.

## CONCLUSION

In summary, the results of the current study demonstrate that RM is sufficient for evaluation of direct smears from FNABs for adequacy and cytologic diagnosis, and validate the feasibility of RM as a tool for remote on-site assessment of FNABs. By extension, the results also raise the possibility that RM is potentially useful for consultations, quality assurance, and teaching activities involving evaluation of FNABs.
